# A comparative study of receptor interactions between SARS-CoV and SARS-CoV-2 from molecular modeling

**DOI:** 10.1007/s00894-022-05231-7

**Published:** 2022-09-08

**Authors:** Hien T. T. Lai, Ly H. Nguyen, Anh D. Phan, Agata Kranjc, Toan T. Nguyen, Duc Nguyen-Manh

**Affiliations:** 1 Key Laboratory for Multiscale Simulation of Complex Systems, VNU University of Science, Vietnam National University, 334 Nguyen Trai street, Hanoi, 11416 Vietnam; 2 Faculty of Materials Science and Engineering, Phenikaa Institute for Advanced Study, Phenikaa University, Hanoi, 12116 Vietnam; 3 Institute of Neuroscience and Medicine (INM-9)/ Institute for Advanced Simulation (IAS-5), Forschungszentrum Jülich, Jülich, 52428 Germany; 4 Laboratoire de Biochimie Théorique, UPR 9080 CNRS, Université de Paris, 13 rue Pierre et Marie Curie, F-75005 Paris, France; 5 Institut de Biologie Physico-Chimique-Fondation Edmond de Rotschild, SL Research University, 75005 Paris, France; 6 CCFE, United Kingdom Atomic Energy Authority, OX14 3DB Abingdon, UK

**Keywords:** SARS-CoV, SARS-CoV-2, RBD-ACE2, Molecular modeling

## Abstract

The pandemic of COVID-19 severe acute respiratory syndrome, which was fatal for millions of people worldwide, triggered the race to understand in detail the molecular mechanisms of this disease. In this work, the differences of interactions between the SARS-CoV/SARS-CoV-2 Receptor binding domain (RBD) and the human Angiotensin Converting Enzyme 2 (ACE2) receptor were studied using *in silico* tools. Our results show that SARS-CoV-2 RBD is more stable and forms more interactions with ACE2 than SARS-CoV. At its interface, three stable binding patterns are observed and named red-K31, green-K353 and blue-M82 according to the central ACE2 binding residue. In SARS-CoV instead, only the first two binding patches are persistently formed during the MD simulation. Our MM/GBSA calculations indicate the binding free energy difference of about 2.5 *kcal*/*mol* between SARS-CoV-2 and SARS-CoV which is compatible with the experiments. The binding free energy decomposition points out that SARS-CoV-2 RBD–ACE2 interactions of the red-K31 ($-23.5~\pm ~0.2~kcal/mol$) and blue-M82 ($-9.1~\pm ~0.1~kcal/mol$) patterns contribute more to the binding affinity than in SARS-CoV ($-1.8~\pm ~0.02~kcal/mol$ for red-K31), while the contribution of the green-K353 pattern is very similar in the two strains ($-17.8~\pm ~0.2~kcal/mol$ and $-22.7~\pm ~0.1~kcal/mol$ for SARS-CoV-2 and SARS-CoV, respectively). Five groups of mutations draw our attention at the RBD–ACE2 binding interface, among them, the mutation –PPA469-471/GVEG482-485 has the most important and favorable impact on SARS-CoV-2 binding to the ACE2 receptor. These results, highlighting the molecular differences in the binding between the two viruses, contribute to the common knowledge about the new corona virus and to the development of appropriate antiviral treatments, addressing the necessity of ongoing pandemics.

**Supplementary Information:**

The online version contains supplementary material available at 10.1007/s00894-022-05231-7.

## Introduction

The COVID-19 disease that has rapidly spread from the Chinese Wuhan city to the rest of the world is caused by the 2019 novel coronavirus (2019-nCoV) named also the severe acute respiratory syndrome corona virus 2 (SARS-CoV-2) [1]. The sequencing of its genome revealed that it is closely related to other coronaviruses, such as severe acute respiratory syndrome coronavirus (SARS-CoV) and Middle East respiratory syndrome coronavirus (MERS-CoV), which have killed hundreds of people in the last two decades [2, 3].

**Fig. 1:**
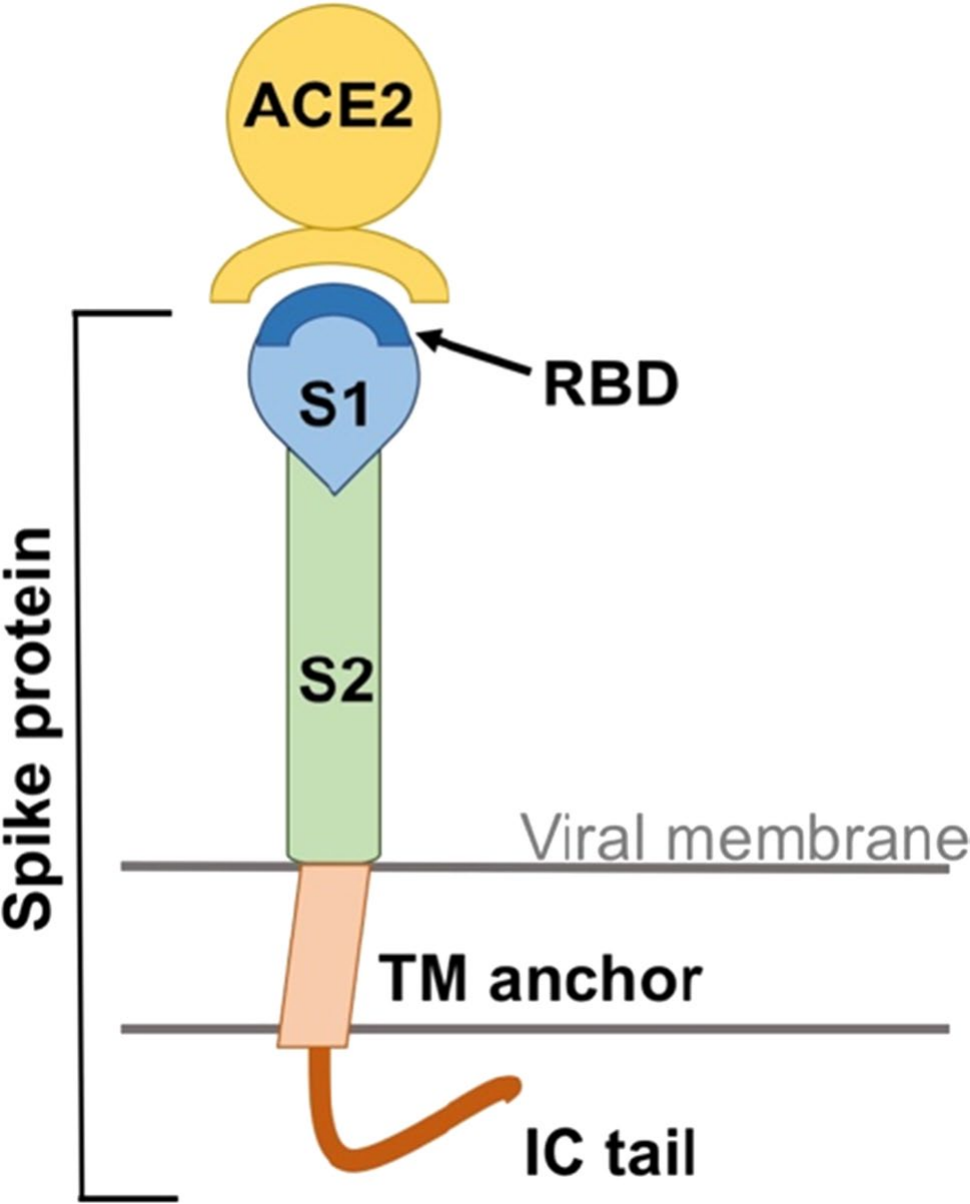
A schematic view of the coronavirus spike protein. The spike protein bears a large ectodomain that is composed of a subunit S1 which has at the top the Receptor Binding Domain (RBD) and the subunit S2. It further features the trans-membrane anchor (TM anchor) and the intracellular tail (IC tail). ACE2 is the human Angiotensin Converting Enzyme 2, the main host entry receptor for SARS-CoV and SARS-CoV-2

Coronaviruses are enveloped, positive sense, single-stranded RNA viruses that carry on their surface spike-like projections giving it a crown-like appearance under the electron microscope; hence the name coronavirus [4]. These spikes (S) enable the fusion between viral and host membranes and are essential for the beginning of the enveloped virus infection [5, 6]. They are composed of a large ectodomain, a trans-membrane anchor and a short intracellular tail (Fig. 1). The ectodomain consists of a receptor-binding subunit S1 and a membrane-fusion subunit S2 which are crucial for binding of the virus to the host cell surface and for entry of the viral genomes into the target cell, respectively [7–11]. The S1 subunit bears the Receptor Binding Domain (RBD) that recognizes and binds to the host protein receptor. The cellular entry receptor for SARS-CoV and SARS-CoV-2 is Angiotensin Converting Enzyme 2 (ACE2) [2, 12–16].

The viral RBD represents one of the main possible targets for the development of antiviral drugs, therefore, understanding the binding between the viral RBD and ACE2 is useful for rational drug design. The SARS-CoV and SARS-CoV-2 are closely related, nevertheless, significant differences were reported for their protein structures. Notably, the mutations in the SARS-CoV-2 RBD domain with respect to the SARS-CoV that can impact the binding affinity for the host receptor [14, 17, 18].

The aim of this study is to compare the structural and energetic differences in the binding of the RBDs of SARS-CoV-2 and SARS-CoV to the ACE2 receptor by Molecular Dynamics (MD) simulations and Molecular mechanics with generalized Born and surface area solvation (MM/GBSA) free energy calculations. Three different systems of ACE2 in complex with the SARS-CoV/SARS-CoV-2 RBDs were studied: (i) SARS-CoV RBD in complex with ACE2, Protein Data Bank (PDB) code: 2AJF [19] (ii) SARS-CoV-2 RBD in complex with ACE2, PDB code: 6M0J [20] (iii) a chimeric SARS-CoV-2 RBD in complex with ACE2, PDB code: 6VW1 [18].

The primary structures of the above systems were compared and five groups of mutations draw our attention. By means of MD simulations the most persistent interactions at the RBD-ACE2 interface were identified and consequently grouped into three binding hot-spots. Binding free energies for the RBD-ACE2 complexes were calculated by means of MM/GBSA method and then decomposed in order to calculate the contribution (i) of each RBD-ACE2 interaction persistent for at least 90% of the MD simulation time and (ii) of each mutation in the five mutation groups.

Our study contributes to better understanding of the binding characteristics between the old and the new coronavirus strains, assisting the process to provide a treatment for SARS-CoV-2 and help to control the pandemic.

## Methods

The RBD - ACE2 complexes were obtained from the RCSB PDB database with IDs: 6M0J [20] for the new SARS-CoV-2 virus, 6VW1 [18] for its chimera, and 2AJF [19] for the SARS-CoV virus. For easy identification, these systems were named 6VW1, 6M0J and 2AJF throughout the article, respectively. The 6VW1 and 2AJF systems contain two complexes of the ACE2 receptor (chains A and B) and the viral RBD domain (chains E and F). While the 6M0J contains only one complex of the ACE2 receptor (chain A) and the viral RBD domain (chain E). In our studies, we kept for each system only one complex of ACE2 (chain A) and the viral RBD (chain E) in order to better compare them to one another. Another reason we decided to continue only with chains A and E is that the complex of chains B and F is about 180$^{\circ }$ rotation symmetry of the complex of chains A and E. This is an artifact of protein crystallization, not actual arrangement of the complexes in real biological system.

There are some notable structural elements in these complexes that need special care when setting up the simulation systems such as the disulfide bonds between various pairs of cysteine residues; the N−linked glycosylations of various asparagine residues; and the Zn-Glu$_{2}$-His$_{2}$ zinc–finger–like (ZF-like) motif (Supplementary Information file, Table S1). In addition, the experimental structures of ACE2 and RBD have some missing residues in their central parts (D615 for ACE2 and A522 for RBD). These residues were added using homology modeling method MODELLER [21–23].

### Sequence alignment and the choice of mutations

The primary sequences of RBDs from 2AJF, 6VW1 and 6M0J were aligned by means of ClustalW web-server [24] using BLOSUM matrix [25] and then visually analyzed in order to find naturally occurring mutations between the SARS-CoV and SARS-CoV-2 using criteria described in details in the Results Section, Sequence and structural analyses.

### Molecular dynamics (MD) simulation

The initial 2AJF, 6VW1 and 6M0J systems, composed of the chain A (human ACE2) and the chain E (viral RBD), were prepared for MD simulations using CHARMM-GUI web-server [26], and then modified manually to properly describe some particular structural elements (Supplementary Information file, Table S1). The MD simulations [27] were performed by GROMACS/2018.6 software package [28]. Proteins and ions were described by Charmm-36 force-field [29] and glycans by GLYCAM06 force-field [30]. The TIP3P [31] model was used for water molecules. The three systems were solvated, sodium and chlorine ions were added to the system to neutralize the total charges and to set physiological electrolyte concentration of solution at 150 mM NaCl. The simulation box size was chosen so that the proteins in the neighbor periodic box are at least 3 nm apart from each other. Since the electrostatic screening length at 150 mM NaCl concentration is about 7Å, this 3 nm distance is more than enough to eliminate the finite size effect due to the long-range electrostatic interactions among proteins in neighboring simulation boxes. In addition, the box size remains small enough that we can have results from MD simulation in a reasonable time regarding our current computational resources. The total numbers of molecules, residues and atoms for the three simulated systems are listed in Supplementary Information file, Table S2.

The geometry of all three studied systems was optimized by steepest descent minimization performed for 5,000 steps with a maximum force constant value of 1000 kJ/mol/nm. After the geometrical optimization, the systems were equilibrated in NPT ensemble for 1 ns at a timestep of 2 fs. The temperature of 310 K and the pressure of 1 atm were maintained by the Berendsen thermostat and barostat [28]. The Particle Mesh Ewald (PME) method [32] was used to treat the long-range electrostatic interaction with a real space cutoff of 1.2 nm. The van der Waals interactions were also cut off at 1.2 nm, with the appropriate cutoff corrections added to pressure and energy. All hydrogen bonds were constrained by the LINCS method [33].

After the equilibration, 2 $\mu$s MD production run at a timestep of 2 fs was performed for statistics. MD simulation parameters were the same as in the NPT equilibration run but the thermostat was changed to the Nose-Hoover thermostat [34–36] and the barostat to the Parrinello-Rahman barostat [37, 38].

### Analyses

**The structural stability/flexibility** was determined by calculating the Root Mean Square Deviation (RMSD) of the backbone and the Root Mean Square Fluctuations (RMSF) of the C$_{\alpha }$ atoms for each residue. The center of mass (COM) distances were calculated between the two RBD segments and their counterpart ACE2 residues L79–Y83. The two RDB segments are as follows: (i) residues T470–C480, which include VPF_2AJF_ and PD_2AJF_ mutation groups, and (ii) residues F486–Y489 that represent the SARS-CoV-2 blue binding pattern. These calculations were performed using GROMACS scripts [28].

**The buried surface area (BSA)** at the ACE2–RBD interface was calculated as follows, where the solvent accessible surface area (SASA) values were obtained by GROMACS scripts [28]:

1 $$\begin{aligned} \mathbf{BSA} _{interface} = ( \mathbf{SASA} _{ACE2} + \mathbf{SASA} _{RBD} ) - \mathbf{SASA} _{ACE2-RBD} \end{aligned}$$

**Frequencies of the RBD interactions with the ACE2 receptor** were calculated using *in-house* made TCL and AWK scripts (reported in the Supplementary Information file). The RBD residues found within 5 Å of any residue belonging to ACE2 were counted as interacting with each other. Only contacts between heavy atoms were taken into account. The frequency of binding was calculated as the number of frames the two residues interact divided by the total number of frames (2001 frames). Residues interacting for at least 90% of the simulation time are presented in Table 1. The type of interactions (van der Waals, electrostatic, hydrophobic or $\pi$-cation interactions) was defined by visual inspection in VMD [39].

**Table 1:** ACE2-RBD interactions persistent for more than 90% of the 2 $\mu$s simulation time

	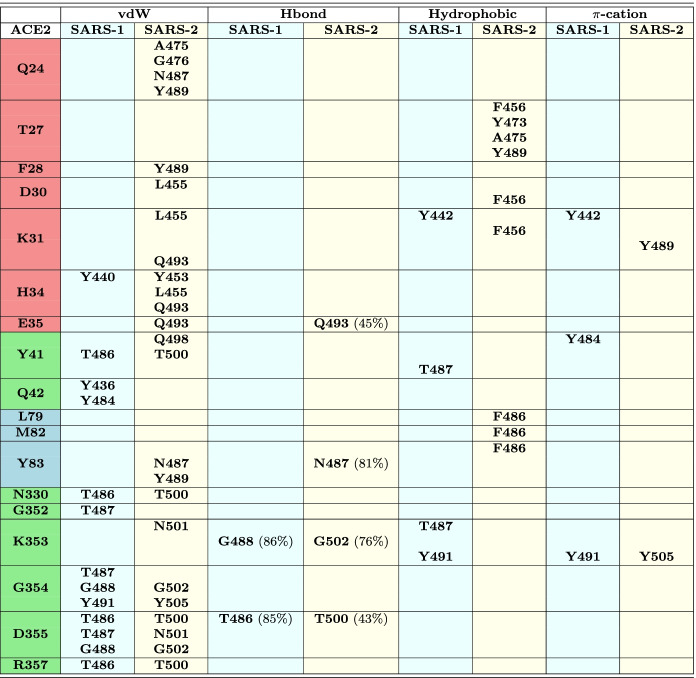

**Fig. 2:**
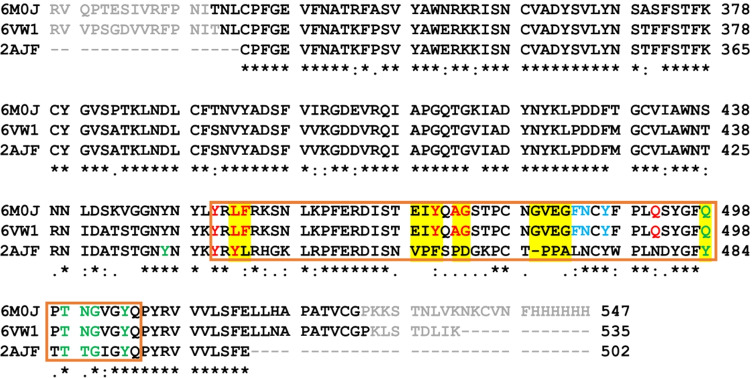
Sequence alignment of viral RBDs structures of SARS-CoV-2 (6M0J), its chimera (6VW1) and SARS-CoV (2AJF). The orange box delineates the Receptor Binding Motif (RBM) in all three systems. Within RBM the chimera 6VW1 residues are the same as in 6M0J sequence, while outside RBM they correspond to 2AJF. Five groups of mutations that draw our interest at the RBD-ACE2 interface are highlighted in yellow. RBD-ACE2 interface residues that interact with each other for at least 90% of the simulation time are shown in red, green and blue, corresponding to red-K31, green-K353 and blue-M82 binding patterns, respectively. The grayed−out residues are those missing in the PDB crystal structures

Systems: SARS-1 – SARS-CoV (2AJF); SARS-2 – SARS-CoV-2 (6M0J & 6VW1). Type of interactions: vdW – van der Waals, Hbond – hydrogen bond, hydrophobic – hydrophobic contacts/interactions, $\pi$-cation interactions. Only Hbonds with persistence of at least 40% of the simulation time are shown. More Hbonds with lower probability are shown in Table S3. Residues in the same line occupy the same position in the sequence alignment (see Fig. 2). Residues in red/green/blue colors belong to the red-K31, green-K353 and blue-M82 binding patterns, respectively. See also Fig. 5

**The hydrogen bonds**: Possible Hbond pairs identified by the above-mentioned TCL and AWK scripts were then analyzed by the *g_hbond* tool in the Gromacs package [28] in order to define their persistence in time. The Hbonds were determined by geometric criteria, namely the distance between the donor and acceptor atoms was at most 3.5 Å, and the angle of the three atoms making up the hydrogen bond was less than 30$^{\circ }$. The Hbond persistence in time was calculated as the number of frames the two residues interact through the Hbond divided by the total number of frames (2001 frames) (Table 1 and Supplementary Information file, Table S3).

**The binding free energy** was calculated for the three RBD-ACE2 complexes, 2AJF, 6M0J and 6VW1, in order to compare the binding affinity of viral RBD for the human ACE2 receptor.

The Molecular mechanics with generalized Born and surface area solvation (MM/GBSA) method was employed using the *mmpbsa.py* script (Eqs 2 & 3) [40, 41]. The resulting binding free energies were scaled down using the scaling factor of 2.45 as proposed for the MM/GBSA method by DasGupta *et al.* [42].

The binding free energies were calculated based on the MD trajectories of 2 $\mu$s, employing 2001 frames sequenced by a time interval of 1 ns (we checked that this is large enough to assure that the energies are non-correlated). The correlation time for our systems was defined by plotting the auto-correlation function of the total energy from the MD simulation code (Supplementary Information file, Fig. S3). The solute dielectric constant was set to 1.0 and the solvent dielectric constant to 80.0. The salt concentration was kept the same as in the MD simulation, notably 0.15M.

In addition, *per-residue* and *per-interaction (i.e. pairwise)* binding free energies (Eq. 3) were calculated using the same method. The per-residue binding free energy was calculated for each residue in the five chosen groups of mutations, and it represents a sum of binding free energies for all interactions the single residue forms with the counterpart ACE2 residues and with its neighbor residues in the viral RBD. In contrast, per-interaction binding free energies were calculated for a single interaction between a chosen residue in RBD and its corresponding residue in ACE2.

2 $$\begin{aligned} \Delta G_{binding} = G_{complex} - (G_{ACE2} + G_{RBD}) \end{aligned}$$

3 $$\begin{aligned} G = E_{vdW} + E_{Elec} + G_{Polar} + G_{non-Polar} \end{aligned}$$

In the equation Eq. 2, the total energy value of each part is a sum of van der Waals (E$_{vdW}$), Electrostatic (E$_{Elec}$), Polar (G$_{Polar}$) and non-Polar (G$_{Non-polar}$) energies of this part (Eq. 3). Here, vdW and electrostatic energies were calculated in vacuum, while the last two terms are the free energies of solvation. The G$_{Polar}$ is the reduction of electrostatic energy when charges are transferred from vacuum into the solution. Since the electric field is reduced by an averaged factor of 8-10 in solution compared to vacuum, E$_{Elec}$ and G$_{Polar}$ nearly cancel each other. For the G$_{non-polar}$ term, the SASA model is used, where it is calculated as the solute−solvent surface area multiplied by a surface tension factor. This surface tension is semi-empirical and set by default to 0.0227 *kJ/mol/*Å$^{2}$. There is a constant $E_{bonded}$ energy of bonded interaction, which cancels out when taking the free energy differences.

The Visual Molecular Dynamics (VMD) program [39] was used for visualization, spatial inspection, surface analyses and for making figures.

## Results

### Sequence and structural analyses

The three RBD structures used in our study in complex with ACE2 are from SARS-CoV (PDB code: 2AJF [19]), and two SARS-CoV-2 viruses (PDB codes: 6VW1 [18] and 6M0J [20]). Residues numbering throughout the manuscript corresponds to SARS-CoV-2 RBD. When SARS-CoV RBD or ACE2 numbering is used, their names are added in the subscript, *2AJF* for SARS-CoV RBD and *ACE2* for the receptor, respectively. The sequences of the human ACE2 receptor are clearly identical in all three studied complexes, there are some small variations in glycosylation (Supplementary Information file, Table S1).

Unlike the human ACE2 receptor, the sequences of coronavirus RBDs differ to varying degrees (Fig. 2). The RBD sequence identity between the SARS-CoV-2 coronavirus (PDB ID: 6M0J) and its chimeric sequence (PDB ID: 6VW1) is 86%, while the sequence identities between SARS-CoV (2AJF) and the two SARS-CoV-2 proteins (6M0J and 6VW1) are 71% and 83%, respectively. Chimera is composed of the SARS-CoV RBD sequence but its Receptor Binding Motif (RBM) sequence (Fig. 2, orange box) is copied from SARS-CoV-2. The C$_{\alpha }$-RMSD between the three structures after the minimization and before the MD simulations are as follows: 2AJF in comparison to 6M0J has the RMSD value of 4.6 Å and in comparison to 6VW1 of 4.2 Å, while the SARS-CoV-2 and its chimera structures differ for 4.9 Å.

We included the chimeric structure in our study in order to understand whether RBM plays the major role causing the difference in ACE2-binding of SARS-CoV-2 or it is the entire RBD. If chimera behaves as SARS-CoV-2 then RBM plays a major role in causing the difference in ACE2-binding with respect to the old strain. If not then the remaining RBD sequence also plays a crucial role.

Since we were interested in major differences in binding between SARS-CoV and SARS-CoV-2, we considered amino acids in the RBM region that are the same in the two SARS-CoV-2 sequences but are not-conserved in SARS-CoV. Among these amino acids, we selected groups of mutations based on the following criteria:

The first group of mutations that draw our attention was:

**–PPA469-471/GVEG482-485**: Two consecutive prolines introduce a certain rigidity into the protein backbone, while the insertion of glycines and the substitutions of the two prolines can lead to higher flexibility of this segment. We hypothesize that GVEG sequence allows to this segment to move closer to the ACE2 receptor with respect to the SARS-CoV and to enable the negatively charged E484 residue to form electrostatic interaction with the positively charged residue K31 of the human ACE2 receptor. We focused then on other SARS-CoV proline mutations found in the RBM sequence alignment and we formed groups of mutations, including one mutation preceding and/or succeeding the proline residues if they introduce an important physico-chemical change. Based on this criteria we ended up with other two groups of mutations including proline residues:
- **VPF458-460/EIY471-473**: This triplet of residues is placed near the interface with the ACE2 receptor, but only the last residue physically interacts with the receptor. The polar and charged glutamic acid instead of hydrophobic valine is favorable in this position since this position is solvent exposed. The rigid proline is exchanged with isoleucine. We hypothesize that this will introduce better flexibility of the protein backbone of this small segment in the new viral structure. Finally, the hydroxyl group of Y473 leads to the possibility for the Hbond interaction with the T27 in ACE2 receptor.
- **PD462-463/AG475-476**: The rigid and bulky, charged amino acids of SARS-CoV (P462 and D463, respectively) are mutated to the smaller and hydrophobic residues (A475 and G476) in the SARS-CoV-2. This change can cause higher flexibility of the loop facilitating that this loop moves closer to the ACE2 receptor and is more prone for binding. The mutation preceding the proline in this group is S461/Q474 (see Fig. 2). We did not include it in the group, since these are both polar residues, very similar in size and do not introduce an important physico-chemical change. Another mutation that draws our attention is mutation YL442-443/LF455-456 for a reason that bulky amino acids are changed for small ones in a reverse order. To this group we added then another SARS-CoV tyrosine mutation of Y484/Q498, for the same reason, bulky polar amino acid is exchanged for polar residue but smaller one:
- **YL442-443/LF455-456**: The first mutation leads to the loss of the aromatic ring and especially of the hydroxyl group of tyrosine, removing the possibility for hydrogen bond with the receptor counterpart residues K31 or E35. In addition, it introduces smaller amino acid in the position of bulkier one. The second mutation is reversed, it exchanges a small hydrophobic amino acid for a bulkier one.
- **Y484/Q498:** this mutation turns the T-stacking hydrophobic interaction between the two tyrosines at the SARS-CoV RBD-ACE2 interface into the electrostatic interaction of SARS-CoV-2 glutamine with the opposing Q42 residue in the ACE2 receptor.

In summary, based on these sequence analyses using the above-mentioned criteria we selected five groups of mutations that may cause the increased backbone flexibility in the RBM motif (when prolines are mutated into other residues) and that introduce physico-chemical changes. We hypothesize that higher backbone flexibility of the part of the SARS-CoV-2 RBM would allow it to adapt better to the receptor binding interface, increasing the number of contacts between the two proteins. The selected five mutation groups, which are located at the RBD-ACE2 binding interface or in its proximity, were studied by MD simulations and Binding free energy calculations. It was out of the scope of this paper to study all mutations found in the SARS-CoV/-2 RBM sequences.

### Structural stability between SARS-CoV-2 and SARS-CoV from MD simulations

Thermal-dynamical movements of the ACE2-RBD complexes in all three studied systems were investigated by means of molecular dynamics (MD) simulation. The three RBD domains differ in length, therefore we included in the analyzes only the so-called *core RBD region* that encompasses residues from 335-CPFGE to VVLSFE-516 (see Fig. 2).

The human ACE2 protein shows similar behavior in all three systems and it is stable with RMSD variations of less than 4 Å with respect to its experimental X-ray structure (Supplementary Information file, Fig. S1). The SARS-CoV-2 RBDs are more stable than SARS-CoV RBD. They deviate from their minimized structures for about 1.5 Å  while SARS-CoV RBD varies for about 3.5 Å (Fig. 4(a)). As a result of these RMSD analyses, in all subsequent equilibrium statistical analyses for the RBD, the first 100 ns of the MD trajectories will be dropped. We calculated as well RMSF for the viral RBDs (Fig. 4(b)). The SARS-CoV-2 RBD domains behave very similarly to each other and are more stable, with less thermal fluctuations, than the SARS-CoV RBD. We describe the most notable differences of the RMSD and RMSF values of the three systems here below.

**Fig. 3:**
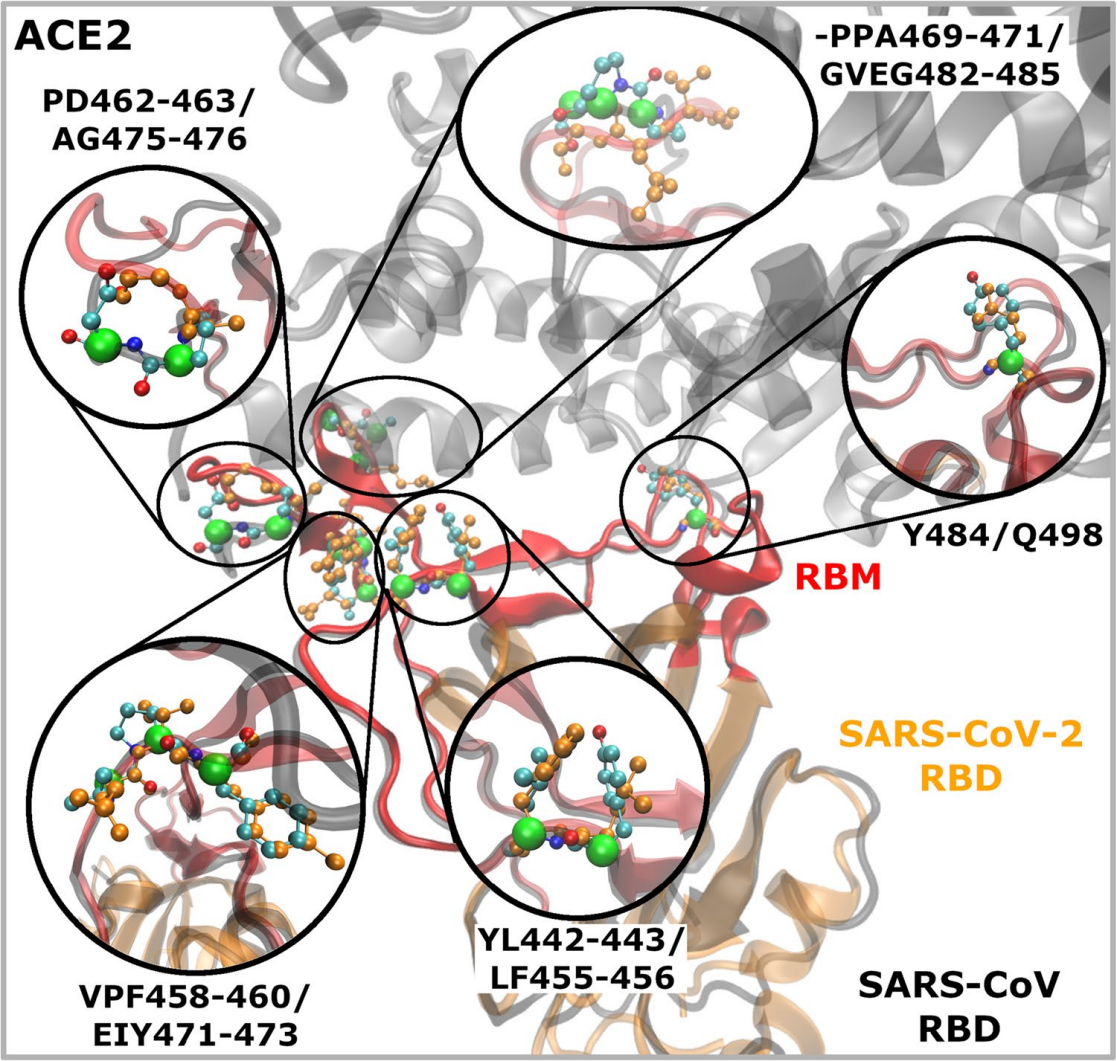
Five groups of mutations at the RBD-ACE2 interface investigated in this study. Annotations are SARS-CoV/SARS-CoV-2. SARS-CoV (PDB ID: 2AJF) and ACE2 are represented in black coil and black cartoon, respectively. SARS-CoV-2 RBD (PDB ID: 6M0J) is depicted in orange cartoon. The receptor binding motif (RBM) of the viral Spike protein is shown in red color. C$_{\alpha }$–green, C–cyan, O–red and N–blue atoms of SARS-CoV residues; the corresponding SARS-CoV-2 amino acids are shown in orange balls and sticks

**Fig. 4:**
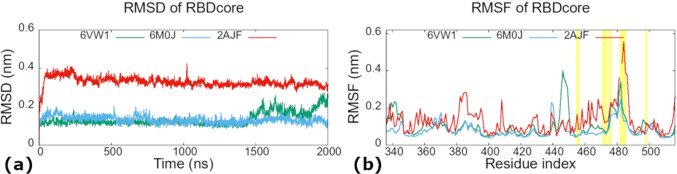
Structural stability of viral SARS-CoV/-2 RBDs throughout 2 $\mu$s of MD simulations. (**a**) The root-mean-square deviation (RMSD) was calculated for the backbone. Both systems of SARS-CoV-2 are represented in green and cyan curves, and SARS-CoV system is shown in red curve. (**b**) The root-mean-square fluctuations (RMSF) were calculated for C$_{\alpha }$ atoms. The yellow vertical lines highlight the fluctuations of the residues present in the five mutation groups. The residue indices of 2AJF were shifted and aligned according to the alignment table shown in Fig. 2, so that the corresponding amino acids of the three viruses are superimposed. Residues indices correspond to SARS-CoV-2 numbering, to obtain the SARS-CoV residue numbers subtract 13

Based on our observations, the higher RMSD value for the 2AJF system is caused by: (i) less compact RBD structure with respect to SARS-CoV-2 due to the shorter central $\beta$-strands composing anti-parallel $\beta$-sheet core of RBD. Higher fluctuations of these parts of SARS-CoV RBD are visible also in the RMSF graph corresponding to the area around SARS-CoV residue positions 327 and 342 (these positions correspond to indices 340 and 355 in the graph, respectively) (Fig. 4(b)); (ii) the missing S-S bridge C378$_{2AJF}$–C511$_{2AJF}$; experimentally resolved 2AJF sequence is shorter than the native one meaning that the C511$_{2AJF}$ was not resolved in the structure. The corresponding S–S bridge in SARS-CoV-2 (C391–C525) connects the C-terminal with the core body of RBD rendering it more stable. Since this interaction is missing in SARS-CoV structure that we used in our MD simulations, its RBD residues 367-387 fluctuate more than in the new viral strain (Fig. 4(b)), indices 380-400 in the graph); (iii) the lower RBM stability in SARS-CoV than in SARS-CoV-2 (Supplementary Information file, Fig. S2). We suggest that this is due to the proline residues present in the RBM section N457–Y475_2AJF_ (Figs. 2 and 3, mutation groups VPF458-460_2AJF_, PD462-463_2AJF_ and –PPA469-471_2AJF_). Prolines render this section of 2AJF more rigid, i.e. less prone to adopt different conformations, therefore, it adapts less well to the binding surface of the ACE2 receptor forming with it less stabilizing interactions (Table 1, and the next subsection) than the RBM of SARS-CoV-2 where these prolines are mutated to other residues (Figs. 2 and 3, and subsection Mutations at the RBD-ACE2 interface). Consequently, this part in 2AJF has higher thermal fluctuations for the residues in the VPF458-460/EIY471-473 and –PPA469-471/GVEG482-485 mutation groups (Fig. 4(b), 2$^{nd}$ and 4$^{th}$ yellow bars). Instead, the thermal fluctuations of residues in the PD462-463/AG475-476 mutation group have similar values in 2AJF and 6VW1, which are higher than in 6M0J (Fig. 4(b), 3$^{rd}$ yellow bar). Furthermore, we observed that this RBM part in 2AJF detaches from the ACE2 receptor for about 600 ns (from 400 ns to 1000 ns) contributing to the higher RMSD values of SARS-CoV with respect to SARS-CoV-2 (subsection Mutations at the RBD-ACE2 interface and Supplementary Information file, Fig. S2).

Finally, the thermal fluctuations of the 2AJF residues in the first mutation group YL442-443/LF455-456 are higher in comparison to the new viral strains, while the residues in the last mutation group Y484/Q498 show very similar fluctuation values (Fig. 4(b), 1$^{st}$ and 5$^{th}$ yellow bars). The impact of these mutations on stability of binding interactions is discussed in subsection Mutations at the RBD-ACE2 interface.

The RMSF graph shows as well an increase of the RMSF values for 6VW1 around residues 440-460 (Fig. 4(b)). The 6VW1 region including this sequence is composed of a long loop, residues A443-Y451. This loop is located just before RBM and does not have direct and stable interactions with ACE2 (its sequence equals to the 2AJF sequence). In the beginning of the MD simulations this loop has the hairpin-like conformation in all three studied systems and is stabilized through five backbone Hbond interactions: D442-O – N448-NH, T444-O – T446-NH, T444-O – G447-NH, T444-NH – F497-O and N448-O – F497-NH. After the first 80 ns of the 6VW1 MD simulations the hairpin-like conformation of this loop changes to a more extended one due to the progressive loss of the Hbonds keeping together the two hairpin sides. In contrast, in the other two systems (2AJF and 6M0J) the Hbonds persist in time and the loop remains in its initial hairpin-like conformation during the full length of the MD simulations. We investigated what could be the possible effect of these changes on 6VW1 RBD. One possible effect we thought about was that there is a well-conserved Y449 located at the C-terminus side of this loop that forms very stable interaction with ACE2 in the 2AJF system, but not in the chimera nor in the 6M0J system. If the loss of interactions between Y449 and ACE2 was a consequence of the changed loop conformation in chimera, then this interaction should be preserved in the 6M0J system since its loop remains stable in the same conformation as in the 2AJF system. But in 6M0J this interaction is not stable either. Different loop conformation in 6VW1 also did not importantly impact the whole RBD, since the conformational change happens at around 80 ns, while the RBD RMSD is very stable throughout MD simulation. We carefully visually inspected the possible effects on 6VW1 RBD due to the loop conformation alteration but we could not identify any well visible effect despite higher local flexibility of this loop.

#### The interaction patterns between RBD and ACE2

Analyses were done to define the type of interactions which maintain the RBD-ACE2 complex and their persistence in simulation time was calculated (Table 1 and Supplementary Information file, Table S3). Only the interactions found for at least 90% of the MD simulation time at the RBD-ACE2 distance of less than or equal to 5 Å were taken into account. From Table 1, it is clear that at this criteria, the new coronavirus RBDs form about twice as much interactions with the ACE2 receptor as the SARS-CoV RBD. Hbond analyses were then done more in detail using standard Hbond criteria for the distance and angle values. We found that SARS-CoV-2 has higher probability to form Hbonds. Nevertheless, two Hbonds in each strain have their lifetime longer than 70% of the simulation time, while others show much lower persistence (Supplementary Information file, Table S3). The bigger number of interactions in SARS-CoV-2 RBD could be associated with its higher binding affinity.

Two binding patterns were identified that are common to both, SARS-CoV and SARS-CoV-2 viruses, while one binding hot-spot was found to be inherent to SARS-CoV-2.

**Fig. 5:**
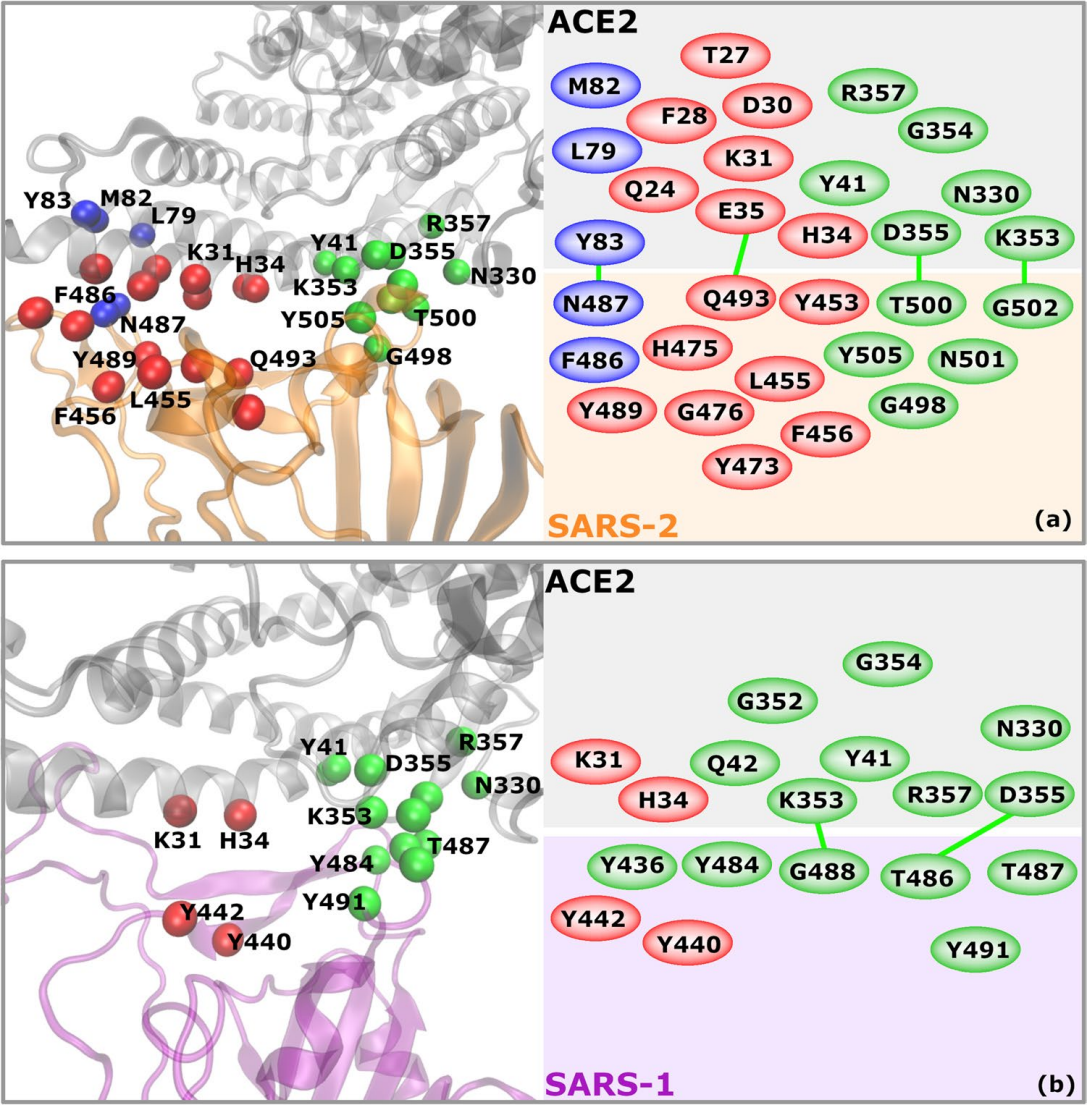
Residues composing the binding patterns at the ACE2 - RBD interface in (**a**) SARS-2 (SARS-CoV-2; 6M0J & 6VW1) and (**b**) SARS-1 (SARS-CoV; 2AJF) systems. Red and green balls represent the red-K31 and the green-K353 binding patterns that are common to both SARS-1 and SARS-2 coronavirus. The blue balls depict the blue-M82 binding pattern that is constantly formed in our MD simulations only in SARS-2. Green lines represent Hydrogen bonds persistent for at least 40% of the MD simulation time. For more details about the interaction pairs and types of interaction see Table 1

The first pattern, which we named red-K31 (Table 1 and Fig. 5, red balls), includes residues at the very beginning of the ACE2 N-terminus, notably Q24_ACE2_, E35_ACE2_, K31_ACE2_ and H34_ACE2_. These four residues interact with RBDs in all three systems, while other residues in this pattern interact constantly only with SARS-CoV-2 RBDs. K31_ACE2_ forms $\pi$-cation interactions with Y442_2AJF_ and with Y489 in SARS-CoV-2. In the latter it interacts also with L455, Q493 and F456. H34_ACE2_ forms vdW interactions with Y440_2AJF_ and with L455 and Q493 in the new strain.

The second pattern, which we named green-K353 (Table 1 and Fig. 5, green balls), consists of ACE2 residues Y41_ACE2_, N330_ACE2_, K353_ACE2_, G354_ACE2_, D355_ACE2_ and R357_ACE2_. They bind with RBD counterpart residues, forming vdW, Hbond, hydrophobic and also $\pi$-cation interactions (Table 1). The latter is formed between K353_ACE2_ and Y491_2AJF_ or Y505 in the new virus strain, respectively.

While these two patterns are found in old and new virus strains alike, the third interaction patch, named blue-M82, (Table 1 and Fig. 5(a), blue balls) was observed almost exclusively in the SARS-CoV-2. It is formed between the ACE2 residues L79_ACE2_, M82_ACE2_ and Y83_ACE2_ and their correlative residues in RBD. The first two residues form hydrophobic interactions with the F486, while the last one makes vdW interactions with N487 and Y489 in addition to the parallel-displaced $\pi$-stacking interaction with F486. In the 2AJF system, only one interaction appears in this pattern (Y475_2AJF_–Y83_ACE2_) that persists for 70% of the simulation time not reaching our criteria of persistence of at least 90%. We observed indeed, as already said above, that 2AJF RBD detaches from the ACE2 during the MD simulation (between around 400 ns and 1000 ns, Supplementary Information file, Fig. S2) and we see here that it detaches exactly at the location of the third binding pattern. We discuss this fact more in detail in the next subsection.

#### Mutations at the RBD-ACE2 interface

We identified and focused our analyses on five groups of mutations: YL442-443/LF455-456, VPF458-460/EIY471-473, PD462-463/AG475-476, –PPA469-471/GVEG482-485, Y484/Q498 (Fig. 2). In the first mutation group YL442-443/LF455-456, Y442_2AJF_, L455 and F456 interact steadily with the ACE2 receptor (Table 1). The number of vdW and hydrophobic interactions in SARS-CoV-2 compensates well for the loss of $\pi$-cation interaction present in SARS-CoV between Y442_2AJF_ and K31_ACE2_. We have not observed stable Hbonds between Y442_2AJF_ and K31_ACE2_ or E35_ACE2_.

The constant interactions were observed between ACE2 and the SARS-CoV-2 residues in the second, the third and the fourth set of mutations, while they are absent in SARS-CoV (Table 1).

**Fig. 6:**
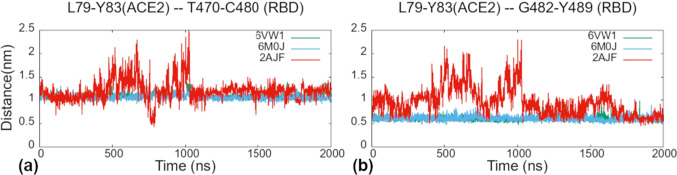
The center of mass (COM) distance between SARS-CoV-2 T470–C480 (N457–C467 in SARS-CoV) loop (a) or G482–Y489 loop (P469–Y475 in SARS-CoV) (b) and their counterpart ACE2 residues L79–Y83 belonging to the blue pattern during the 2 $\mu$s simulation time. The green and blue lines are for 6VW1 and 6M0J systems, respectively, and the red line is for the 2AJF system

These three sets of mutations are located in the RBM section, residues T470 – Y489. We calculated the center of mass (COM) distance of the two segments of this RBM part with respect to its ACE2 counterpart (Fig. 6). The first segment includes residues T470 – C480, and VPF458-460/EIY471-473, PD462-463/AG475-476 mutation groups (Fig. 2). In the new viral strains the center of mass of this segment shows throughout the MD simulation small and steady fluctuations around the average distance of 10.7 Å from ACE2. In contrast, the same segment in SARS-CoV fluctuates more and at around the average distance value of 12.1 Å (Fig. 6(a)).

The second segment, residues G482 – Y489, for which we calculated the COM distances includes –PPA469-471/GVEG482-485 mutation group (Fig. 2). In SARS-CoV-2 this segment fluctuates steadily around the average distance value of 6.1 Å  while it fluctuates much more in the old strain and around higher average distance value of 9.6 Å (Fig. 6(b)).

We observe that the three mutation groups including proline residues in the RBM section T470 – Y489, cause the difference in binding between the two viruses. As we and others [18] suggested, the substitution of rigid prolines for other residues makes the backbone more flexible, i.e. more prone to adopt different conformations and consequently to adapt better to the ACE2 binding surface. In turn, more residues in this section can form stable interactions with ACE2 rendering it more stable in time what reflects through more stable RMSD, RMSF and COM values with respect to the SARS-CoV strain.

Indeed, mutations in the first segment lead to the regular interactions between Y473, A475 and G476 residues and ACE2 (Table 1), while the interactions of the same SARS-CoV segment with ACE2 are less constant (their persistence in time is less than 90%).

The –PPA469-471/GVEG482-485 mutation in the second segment has even stronger impact on SARS-CoV-2 binding to ACE2. While residues in this group do not directly interact with ACE2, residues following them — F486, N487 and Y489 — are brought closer to the ACE2 receptor and new interactions, very stable in time are established. Very interestingly, residues F486, N487 and Y489, form the third binding pattern, unique to the SARS-CoV-2, that we named blue-M82 (subsection The interaction patterns between RBD and ACE2, Table 1, Fig. 5, blue color).

As already mentioned above, the presence of a group of prolines that are located very close to one another in this SARS-CoV section of RBM (residues Y440- E502) leads to less flexible backbone and consequently to less conformational freedom. As seen from our results, this leads to less persistent contacts between this SARS-CoV RBM section and ACE2 provoking a well visible detachment of this part of RBD from the receptor for about 600 ns during the MD simulation (from 400 ns to 1000 ns) (Fig. 6 and Supplementary Information file, Fig. S2(b)).

Finally, in the fifth mutation group, Y484/Q498, both residues constantly interact with the ACE2 receptor (Table 1).

Based on the results from these qualitative analyses, we extended our study with binding free energy calculations: (i) to evaluate the binding affinity between the two SARS strains; (ii) to calculate binding free energy contributions of RBD-ACE2 interactions defined in Table 1 and (iii) to assess the impact of mutations on RBD-ACE2 binding.

### Binding free energy calculations

Binding free energies were calculated for the three RBD-ACE2 complexes using the Molecular mechanics with generalized Born and surface area solvation (MM/GBSA) method [40] (see Methods, equations 2 and 3). We performed a number of tests employing Molecular mechanics/Poisson-Boltzmann Surface Area (MM/PBSA) and Molecular mechanics/generalized Born Surface Area (MM/GBSA) methods, applying different solute dielectric constants (1.0, 2.0 and 4.0) and salt concentrations of 0.0 M and 0.15 M (results not shown). We found out that for our systems the MM/GBSA method, with solute dielectric constant of 1.0 results in binding free energies that are the most compatible with the experiments [18, 43, 44]. Salt concentrations did not have important impact on the resulting binding free energies, therefore 0.15 M was used as in the MD simulation. It is well known that MM/PBSA and MM/GBSA usually overestimate the absolute binding free energy, therefore the resulting binding free energies were scaled down using the scaling factor of 2.45 as proposed for the MM/GBSA method by DasGupta *et al.* [42]. So obtained binding free energy results were used to estimate the qualitative differences between the two COVID viruses, not the absolute values of the binding free energies.

Based on our calculations (Supplementary Information file, Table S4) the electrostatic, vdW and hydrophobic interactions are in favor of SARS-CoV-2. The MM/GBSA calculated and scaled down binding free energy for SARS-CoV-2 is of $-13.50~\pm ~0.09$ *kcal*/*mol*. The binding free energy was similar for the chimeric structure, but a little less favorable for the SARS-CoV complex ($-12.56~\pm ~0.07~kcal/mol$, $-10.96~\pm ~0.13~kcal/mol$, respectively). Our result that SARS-CoV-2 binds for about 2.5 *kcal*/*mol* more strongly to the ACE2 receptor than SARS-CoV is much more compatible with the experiments [18, 43, 44] than the binding affinity differences, as high as $20-30~kcal/mol$, reported previously by other similar computational studies [45–47].

We suppose that the major differences between the two viruses lie in the specific types of interactions among the individual amino acids which we analyze in detail here below, with particular focus on mutations of our interest.

#### Pairwise decomposition of binding free energy

We decomposed SARS-CoV and SARS-CoV-2 binding free energies into a *per-interaction* (i.e. per-wise) binding free energy in order to identify interactions reported in Table 1 that contribute the most to the virus-receptor binding. The binding free energies reported here were calculated for a single interaction between the residue in RBD and the corresponding residue in ACE2 (Table 2).

**Table 2:** Binding free energy (in *kcal/mol*) per-interaction for all RBD-ACE2 interactions persisting for at least 90% of the simulation time

	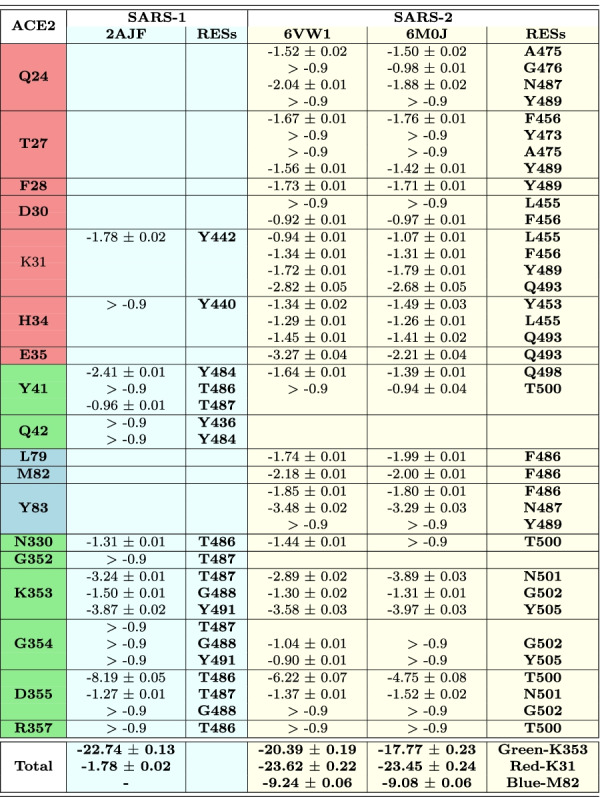

ACE2 residues highlighted in red/green/blue background colors and the SARS-1/2 residues listed in the same lines belong to the red-K31, green-K353 and blue-M82 binding patterns, respectively. The total binding free energy and the standard errors of the mean are reported for each residue and each pattern; values “> -0.9” were excluded; SARS-1, SARS-CoV; SARS-2, SARS-CoV-2

In the red-K31 pattern, SARS-CoV has two stable interactions, K31_ACE2_–Y442_2AJF_ and H34_ACE2_–Y440_2AJF_, but only the first one has a strong binding free energy contribution. The $\pi$-cation interaction was expected between K31_ACE2_–Y442_2AJF_, though the binding free energy value is not as high as in other similar interactions. A possible explanation could be non-optimal spatial position of the two residues for the formation of a strong $\pi$-cation interaction. SARS-CoV-2 system instead has five residues that strongly interact with K31_ACE2_ and H34_ACE2_. In addition, other stable, strong interactions are formed between SARS-CoV-2 RBM and ACE2 receptor in this pattern, notably with Q24_ACE2_, T27_ACE2_, F28_ACE2_, D30_ACE2_ and E35_ACE2_ (Table 2). The strongest interactions observed in this pattern in SARS-CoV-2 are Hbonds between Q24_ACE2_–N487, E35_ACE2_–Q493 and K31_ACE2_–Q493. The Hbond of Q493 was identified as intermittent between K31_ACE2_ and E35_ACE2_ during the MD simulation.

The total binding free energy contribution of the red-K31 pattern is much bigger in SARS-CoV-2 ($-23.45~\pm ~0.24~kcal/mol$) than in SARS-CoV ($-1.78~\pm ~0.02~kcal/mol$) (Table 2), giving to SARS-CoV-2 an important advantage for binding to the human receptor, with respect to the old virus.

In the green-K353 pattern, the old and the new virus have very similar number of stable interactions, as well as the values of binding energies between these interactions. This may indicate the great importance of this binding pattern for the SARS virus entry into the human host regardless of the strain. SARS-CoV has few more constant interactions in this pattern than SARS-CoV-2 (Table 1). The strongest interactions within this pattern are $\pi$-stacking between Y41_ACE2_–Y484_2AJF_, Hbond between K353_ACE2_–T487_2AJF_/N501, $\pi$-cation interactions between K353_ACE2_–Y491_2AJF_/Y505 and Hbond between D355_ACE2_–T486_2AJF_/T500. The green-K31 pattern total binding free energy is a little in favor of SARS-CoV ($-22.74~\pm ~0.13~kcal/mol$) in comparison to the new virus ($-17.77~\pm ~0.23~kcal/mol$) (Table 2).

As already said above, these two patterns were found in both, old and new SARS viruses. The third binding pattern, blue-M82, with the interactions persistent for more than 90% of the simulation time, was instead observed only in SARS-CoV-2. This pattern gives an important additional contribution of $-9.08~\pm ~0.06~kcal/mol$ (Table 2) to the total binding free energy between SARS-CoV-2 and its host receptor. Interactions within this pattern are mostly hydrophobic and an Hbond with 81% of persistence in simulation time is formed between Y83_ACE2_ and N487.

The total pairwise binding free energy contribution of the three binding patterns is of $-50.30~\pm ~0.53~kcal/mol$ in SARS-CoV-2 and of $-24.52~\pm ~0.15~kcal/mol$ in SARS-CoV (Table 2).

#### Effect of mutations on RBD-ACE2 binding free energy

The binding free energies for SARS-CoV-2 and SARS-COV were decomposed into a *per-residue* based binding free energy for the five groups of mutations to evaluate the effect that single RBM mutation has on viral binding (Table 3). They were calculated as a sum of binding free energies for all interactions a single residue in this group forms with counterpart ACE2 residues and with its neighboring residues in the viral RBM.

**Table 3:** Per-residue binding free energy (in *kcal/mol*) for each residue in the mutation group of interest

	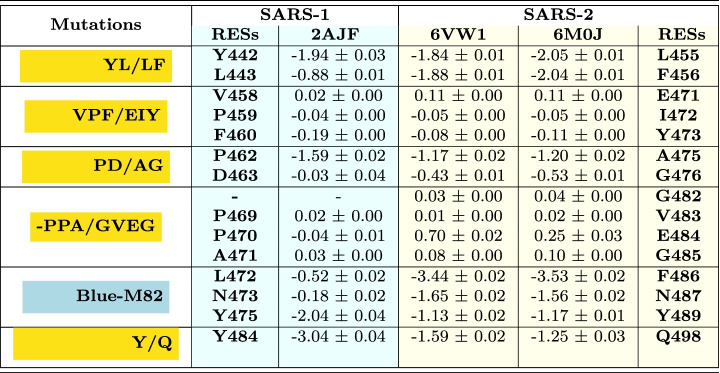

The five mutation groups are highlighted in yellow; errors represent standard errors of the mean; SARS-1, SARS-CoV; SARS-2, SARS-CoV-2

In the first group, YL442-443/LF455-456, the exchange of bulky Y442_2AJF_ side chain with the polar-OH group, for smaller and hydrophobic L455 does not have an important effect on binding free energy since the original hydrophobic interaction is preserved. In contrast, exchanging small L443_2AJF_ for bigger F456 induces a $\Delta G_{binding}$ increase for about 1 *kcal*/*mol* (Table 3). Indeed, L443_2AJF_ interacts few with ACE2. But, F456 – due to its bulkier side chain – becomes stably sandwiched between T27_ACE2_-CH3 group and the K31_ACE2_ side chain -CH2- groups forming hydrophobic interactions.

The second, the third and the fourth groups of studied mutations do not form strong interactions with the ACE2 receptor as it can be seen from their negligible $\Delta G_{binding}$, except P462/A475. Both residues form hydrophobic interactions with -CH2-groups of Q24_ACE2_ and T27_ACE2_. Residues Y473, A475 and G476 form weak hydrophobic and vdW interactions with ACE2 and exhibit similar $\Delta G_{binding}$ as the corresponding residues in SARS-CoV, but they constantly interact with ACE2 for more than 90% of the simulation time, while the old strain residues do not.

The fourth group of mutations, –PPA469-471/GVEG482-485, makes the backbone of this segment in SARS-CoV-2 more flexible, since rigid proline residues were exchanged with small and flexible glycines. As already suggested above, due to the higher flexibility this segment comes closer to ACE2 and remains in its vicinity throughout the MD simulation time (Fig. 6(b)) enabling residues F486, N487 and Y489, which follow immediately the GVEG motif, to constitute the blue-M82 binding pattern with strong binding affinity for the ACE2 receptor (Table 3). In contrast, the same 2AJF segment fluctuates much more and remains more distant from ACE2 during almost all MD simulation time (Fig. 6(b)). Among the three residues, F486 and N487 have bigger contribution to the $\Delta G_{binding}$ than the same residues in SARS-CoV with the values of $-3.53~\pm ~0.02~kcal/mol$ and $-1.56~\pm ~0.02~kcal/mol$, respectively. They form hydrophobic, $\pi$-stacking and vdW interactions with L79_ACE2_, M82_ACE2_ and Y83_ACE2_. The $\Delta G_{binding}$ of the corresponding residues in SARS-CoV is negligible, pointing to the importance of these residues for SARS-CoV-2 binding. Indeed, F486 that corresponds to L472_2AJF_ with its bulkier side chain fits like a tenon in mortise composed of L79_ACE2_, M82_ACE2_ and Y83_ACE2_. The third residue in this group is Y489 ($-1.17~\pm ~0.01~kcal/mol$) and its corresponding residue in SARS-CoV is Y475_2AJF_, which has more favorable binding affinity ($-2.04~\pm ~0.04~kcal/mol$). However, during our MD simulations it has lower frequency of interactions with the receptor than Y489 (70.1% vs 97.8%, respectively). In addition, the total contribution of binding affinity of these three SARS-CoV-2 residues in the blue-M82 pattern is greater than in SARS-CoV ($-6.26~\pm ~0.05~kcal/mol$ *vs* $-2.74~\pm ~0.08~kcal/mol$, respectively; Table 3) adding an important binding spot in favor of the new virus strains.

In the fifth group, the mutation Y484/Q498 is less favorable for binding ($-3.04~\pm ~0.04$ *kcal*/*mol* *vs* $-1.25~\pm ~0.03~kcal/mol$, respectively). Indeed, Y484_2AJF_ forms parallel $\pi$-stacking interactions with Y41_ACE2_ and intermittent Hbond with Q42_ACE2_, while Q498 interacts only through weaker vdW interactions with Y41_ACE2_.

Our results are in line with those reported by Spinello *et al.* [45]. We all found by means of MM/GBSA *per-residue* decomposition the favorable ACE2 binding free energy for same SARS-CoV-2 residues that we report in Table 3. And we all found that P462_2AJF_ has favorable binding for about 0.4 *kcal/mol* with respect to its counterpart SARS-CoV-2 residue A475. However, P462 does not interact constantly with ACE2 receptor while A475 it does. In contrast, they report about much stronger binding free energy for Q498 while we observed more favorable binding free energy for SARS-CoV residue Y484_2AJF_.

## Discussion

Within a short time, a novel SARS-CoV-2 virus spread around the Earth, affecting health care, social and economic life in more than 250 countries. It immediately caught a great scientific interest, and different studies focused on binding of SARS-CoV and SARS-CoV-2 to the human ACE2 receptor, found to be a major entry for the novel virus into humans [13, 14, 18].

We have run MD simulation of 2 $\mu$s for the three complexes: SARS-CoV (2AJF)–ACE2, SARS-CoV-2 (6M0J)–ACE2 and for the chimeric SARS-CoV-2 (6VW1)–ACE2 in order to compare binding between the old and the new coronavirus. We studied as well different groups of mutations existent at the RBD-ACE2 interface.

By means of the MM/GBSA method [40, 41] we estimated about 2.5 *kcal*/*mol* stronger binding affinity of SARS-CoV-2 with respect to the old strain. Our results are thus compatible with the very small experimental binding free energy difference (calculated from the experimentally reported K_D_ values [18, 43, 44]) of $0.8-1.4~kcal/mol$ in favor of the SARS-CoV-2. On the contrary, previous computational studies based on MM/PBSA– or MM/GBSA–techniques estimated that the binding of the new virus would be as much as $20-30~kcal/mol$ stronger with respect to the old one [45–47], incompatibly with experiments.

Regarding the stability, we observed the most notable difference in the RBM motif, which is much more stable in SARS-CoV-2 than in SARS-CoV. We furthermore observed that the long loop connecting $\beta$5 and $\beta$6 in SARS-CoV RBD (P459_2AJF_–Y475_2AJF_, corresponding to $\beta$7 and $\beta$8 in SARS-CoV-2), which interacts with the N-terminal part of ACE2, detaches from the receptor for about 600 ns (from 400 ns to 1000 ns). The loop then approaches again back to ACE2. Intermittent detachment of this SARS-CoV loop from the ACE2 receptor was reported also by Pavlova *et al.* [48].

According to our observations, the partial detachment in SARS-CoV is caused because of the rather rigid RBM segment N457 – 475_2AJF_ that includes four proline residues while the corresponding SARS-CoV-2 segment T470 — Y489 has only one. It is known that mutations may have not only direct but also indirect effects on binding. In our case the three mutation groups in this segment, VPF458-460/EIY471-473, PD462-463/AG475-476 and –PPA469-471/GVEG482-485 have indirect impact on SARS-CoV-2 binding through alteration of this segment conformation. The non-proline residues allow for better flexibility of this SARS-CoV-2 RBM section, leading to more conformational freedom thus this segment can adapt better to the ACE2 binding interface. While VPF458-460_2AJF_ and PD462-463_2AJF_ scarcely interact with ACE2, the corresponding SARS-CoV-2 residues Y473, A475 and G476 form constant interactions with ACE2. Even stronger indirect effect on binding has –PPA469-471/GVEG482-485 mutation.

As already suggested in Shang *et al.* [18], the GVEG482-485 sequence enables $\beta$7–$\beta$8 loop in SARS-CoV-2 RBD to move closer and to adapt better to the surface of the ACE2 receptor thus allowing to the surrounding residues to form more stable interactions. Notably, residues that follow the GVEG482-485 motif strongly and persistently interact with the blue-M82 binding pattern (five interactions, stable for 99% of the simulation time in SARS-CoV-2 *vs* one interaction persistent for 70% of the simulation time in SARS-CoV). Our calculations show that the interactions within the blue-M82 hot-spot (residues L79_ACE2_, M82_ACE2_ and Y83_ACE2_) contribute to the $\Delta G_{binding}$ in SARS-CoV-2 $-9.08~\pm ~0.06~kcal/mol$.

In addition to the blue-M82 hot-spot, the red-K31 binding pattern contributes strongly to the $\Delta G_{binding}$ in SARS-CoV-2 with respect to SARS-CoV strain. This pattern, observed in SARS-CoV and SARS-CoV-2 alike, includes ACE2 residues Q24, T27, F28, D30, K31, H34, and E35. We found that SARS-CoV-2 has eight persistent interactions within this patch, while SARS-CoV has only two. This difference is partially a consequence of the detachment in the SARS-CoV that we described above. Therefore, there is a notable difference in the contribution of the red-K31 binding pattern to the $\Delta G_{binding}$ that is of $-23.45~\pm ~0.24~kcal/mol$ for SARS-CoV-2 and of $-1.78~\pm ~0.02~kcal/mol$ for SARS-CoV.

The third, green-K353 binding patch, comprises ACE2 residues Y41, Q42, N330, K353, G354, D355 and R357 and is placed at the opposite site of the blue-M82 patch. This binding pattern is equally stable in both strains, having the same number of interactions and similar total $\Delta G_{binding}$ contribution ($-17.77~\pm ~0.23~kcal/mol$ for SARS-CoV-2 and $-22.74~\pm ~0.13~kcal/mol$ for SARS-CoV).

Jafary *et. al.* identified four hot-spot ACE2 residues (Q24–D38, Y41-Q42, M82–Y83 and N330–R357) playing important role in interactions of SARS-CoV and SARS-CoV-2 [46]. Our results are in line with theirs, but we grouped the hot-spot residues, according to their spatial arrangement into three groups, as described above. Our results are in line also with the findings of other groups that worked on identification and on comparison of critical interactions between the old and the new coronaviruses, though they do not necessarily group the ACE2 residues into the binding patterns [47–50].

Five groups of mutations (YL442-443/LF455-456, VPF458-460/EIY471-473, PD462-463/AG475-476, –PPA469-471/GVEG482-485 and Y484/Q498) were studied, in order to see how do they impact binding to ACE2. The mutation YL/LF, especially exchanging L443_2AJF_ to F456 has a favorable impact on the binding affinity. L443_2AJF_, being small does not exhibit steady interactions with the ACE2 receptor, while bulkier F456 interacts with three counterpart residues T27_ACE2_, D30_ACE2_ and K31_ACE2_. Mutations in the second and the third groups, VPF458-460/EIY471-473 and PD462-463/AG475-476, do not exhibit any changes for the binding affinity between SARS RBD and ACE2.

The most important mutation is –PPA469-471/GVEG482-485, which increases the flexibility of SARS-CoV-2 RBD $\beta$7–$\beta$8 loop as already said above. Consequently, residues F486, N486, and Y489 come closer to the ACE2 receptor and constantly interact with the blue-M82 binding pattern. Within these residues the most favorable mutation for binding is L472/F486 ($-0.52~\pm ~0.02~kcal/mol$ *vs* $-3.53~\pm ~0.02~kcal/mol$, respectively). The F486 residue is enveloped by the hydrophobic counterpart residues from the blue-M82 binding patch, L79_ACE2_, M82_ACE2_ and its bulky aromatic side chain is forming stable $\pi$-stacking interactions with Y83_ACE2_. Our observations confirms scenario, which was suggested by Chen *et al.* [51].

And the last mutation Y484/Q498 is less favorable with $-3.04~\pm ~0.04~kcal/mol$ in SARS-CoV vs $-1.25~\pm ~0.03~kcal/mol$ in SARS-CoV-2. Indeed, Y484_2AJF_ is making favorable parallel $\pi$-stacking interaction with Y41_ACE2_ and intermittent Hbond with Q42_ACE2_. While the interactions of Q498 are limited to the weaker, vdW interactions with Y41_ACE2_. In our MD simulations Hbond with Y41_ACE2_ is formed very rarely. Experiments also demonstrated that at this position tyrosine is more adapted and exhibits favorable binding affinity with respect to the glutamine [52].

Our observations are in line with other computational studies focusing on advantages/disadvantages of mutations at the binding interface between the ACE2 receptor and the old and new coronaviruses [45, 48].

Finally, our results point out that chimera behaves very similar to SARS-CoV-2 indicating that RBM plays a major role in causing the difference in ACE2-binding between the old and the new strain and that the body of the RBD has little influence on RBM. There are rather mutations in RBM that impact binding affinity of the virus for the ACE2 receptor.

## Conclusion

In light of many previous studies of RBD-ACE2 complexes and difference from that of SARS-CoV virus, we would like to stress that our work is a comprehensive extension of our preliminary results published in bioRxiv preprint (https://www.biorxiv.org/content/10.1101/2020.04.21.053009v1), when the experimental structure of SARS-CoV-2 RBD (PDB code: 6M0J) was just published (in April 2020).

From that time, several other works appeared investigating similar systems, but they focus mostly on the biochemical aspect of the interactions, specifically on the electrostatics and hydrogen bond formation or comparing the binding free energy contribution changes of a single mutation/interaction. Our manuscript differs from this line of thinking by studying the biophysical aspect of the five main groups of mutations and of each binding pattern at the RBD-ACE2 interface. In our study we show that –PPA469-471/GVEG482-485 mutation triggers an important structural consequence: the substitution of prolines in SARS-CoV by glycines in SARS-CoV-2 makes the SARS-CoV-2 RBD backbone in this region more flexible, allowing it to adapt better to the receptor interface and resulting in formation of the additional persistent binding pattern (blue-M82). This result is in accordance with the experiments [18]. In addition, MD simulations and free energy calculations permit the evaluation and comparison of the impact this mutation has on the stability and on the strength of the interactions between SARS-CoV-2 and ACE2. We calculated that the pattern formed due to this mutation contributes importantly, about 9 *kcal*/*mol* to the binding free energy between SARS-CoV-2 RBD and ACE2.

Three binding patterns persistent in simulation time were found in SARS-CoV-2 (red-K31, green-K353 and blue-M82), but only two of them in SARS-CoV (red-K31, green-K353). The binding free energy difference that we observed between SARS-CoV-2 and SARS-CoV is of about 2.5 *kcal*/*mol*, a result compatible with the experiments.

Our results elucidate relevant information about the RBD-ACE2 binding interface and can assist in design of potential compounds/antibodies for inhibition of viral activities, contributing to provide viral therapy for COVID-19.

## Supplementary Information

Below is the link to the electronic supplementary material.

Supplementary file 1 (PDF 496 KB)

## Data Availability

The MD trajectories generated and analyzed during the current study are available from the corresponding authors on reasonable request.
