# Supplementary material for: A comparative study of receptor interactions between SARS-CoV and SARS-CoV-2 from molecular modeling
Source: J Mol Model. 2022 Sep 8;28(10):305. doi: 10.1007/s00894-022-05231-7 (PMC9453729; doi:10.1007/s00894-022-05231-7)
Supplement: Supplementary file 1 — (PDF 496 KB) [file 894_2022_5231_MOESM1_ESM.pdf]

A comparative study of receptor interactions between SARS-CoV and SARS-CoV-2 from molecular modelling

Hien T. T. Lai[1†], Ly H. Nguyen[1†], Anh D. Phan[2†], Agata Kranjc[3,4,5*], Toan T. Nguyen[1*] and Duc Nguyen-Manh[6]

[1*]Key Laboratory for Multiscale simulation of Complex Systems, VNU University of Science, Vietnam National University, 334 Nguyen Trai street, Hanoi, 11416, Vietnam.
[2]Faculty of Materials Science and Engineering, Phenikaa Institute for Advanced Study, Phenikaa University, Hanoi, 12116, Vietnam.
[3*]Institute for Advanced Simulations (IAS)-5/Institute for Neuroscience and Medicine (INM)-9, Forschungszentrum Jülich, 52428, Jülich, Germany.
[4]Laboratoire de Biochimie Théorique, UPR 9080 CNRS, Université de Paris, 13 rue Pierre et Marie Curie, F-75005, Paris, France.
[5]Institut de Biologie Physico-Chimique-Fondation Edmond de Rotschild, PSL Research University, 75005, Paris, France.
[6]CCFE, United Kingdom Atomic Energy Authority, Abingdon, OX14 3DB, United Kingdom.

*Corresponding author(s). E-mail(s): a.kranjc.pietrucci@fz-juelich.de; toannt@hus.edu.vn;
Contributing authors: laithithuhien_t60@hus.edu.vn; nguyenhaily_t60@hus.edu.vn; anh.phanduc@phenikaa-uni.edu.vn; Duc.Nguyen@ukaea.uk;
†These authors contributed equally to this work.

1 Materials and Methods

1.1 Systems composition

Table S1: Detailed information of various special structural elements of the SARS-CoVs RBD – ACE2 complexes that needed proper care when setting up the systems for MD simulations. ZF–like stands for zinc-finger–like motif.

**Models**			**S–S bridges**	**ZF-like**	**Glycosylation**
**SARS-CoV-2**	**6VW1**	ACE2	C133 - C141<br>C344 - C361<br>C530 - C542	H374<br>E375<br>H378<br>E402	bDMan(1→4)bDGlcNAc(1→4)bDGlcNAc(1→)N90<br>bDMan(1→4)bDGlcNAc(1→4)bDGlcNAc(1→)N546<br>bDGlcNAc(1→4)bDGlcNAc(1→)N53<br>bDGlcNAc(1→4)bDGlcNAc(1→)N322<br>bDGlcNAc(1→)N103
		RBD	C336 - C361<br>C379 - C432<br>C391 - C525<br>C480 - C488		bDMan(1→4)bDGlcNAc(1→4)bDGlcNAc(1→)N343
	**6M0J**	ACE2	C133 - C141<br>C344 - C361<br>C530 - C542	H374<br>E375<br>H378<br>E402	bDGlcNAc(1→)N90<br>bDGlcNAc(1→)N546<br>bDGlcNAc(1→)N322
		RBD	C336 - C361<br>C379 - C432<br>C391 - C525<br>C480 - C488		bDGlcNAc(1→)N343
**SARS-CoV**	**2AJF**	ACE2	C133 - C141<br>C344 - C361<br>C530 - C542	H374<br>E375<br>H378<br>E402	bDMan(1→4)bDGlcNAc(1→4)bDGlcNAc(1→)N90<br>bDGlcNAc(1→)N53<br>bDGlcNAc(1→)N322<br>bDGlcNAc(1→)N546
		RBD	C323 - C348<br>C366 - C419<br>C467 - C474		bDGlcNAc(1→)N343

In our MD simulations, all ACE2 receptors and RBD in the 2AJF system have three S–S bridges, while RBDs in the 6M0J and 6VW1 systems have four of them. The ACE2 receptor has the zinc ion coordinated by the two glutamate and two histidine residues (Table S1) that need proper protonation [1]. The two glutamate residues in this ZF–like motif, $E375_{ACE2}$ and $E402_{ACE2}$, were kept negatively charged, while the two histidine residues, $H374_{ACE2}$ and $H378_{ACE2}$, were kept neutral with one hydrogen atom bound to N_δ of the histidines. The zinc ion was charged 2^+.

Both, ACE2 and RBD have sugars of different sizes attached to the specific asparagine residues (Table S1). The ACE2 receptor in the 6VW1 system is most heavily glycosylated with $N90_{ACE2}$ and

$N546_{ACE2}$ bearing each an amino trisaccharide consisting of beta-D-mannopyranosyl, 2-acetamido-beta-D-glucopyranosyl and 2-acetamido-D-glucopyranosyl molecules joined sequentially by (1→4) glycosidic linkages (bDMan(1→4)bDGlcNAc(1→4)bDGlcNAc(1→)). The $N53_{ACE2}$ and $N322_{ACE2}$ residues in this system carry an amino disaccharide composed of 2-acetamido-beta-D-glucopyranosyl and 2-acetamido-D-glucopyranosyl moieties joined sequentially by (1→4) glycosidic linkages (bDGlcNAc(1→4)bDGlcNAc(1→)). Finally, the amino monosaccharide 2-acetamido-beta-D-glucopyranosyl (bDGlcNAc(1→)) is attached to $N103_{ACE2}$. The ACE2 residues $N90_{ACE2}$, $N546_{ACE2}$ and $N322_{ACE2}$ of the 6M0J system and $N53_{ACE2}$, $N322_{ACE2}$, $N546_{ACE2}$ of the 2AJF system are all glycosylated with the monosaccharide bDGlcNAc(1→), while $N90_{ACE2}$ in the latter system is linked to the trisaccharide bDMan(1→4)bDGlcNAc(1→4)bDGlcNAc(1→). In the RBD domain only N343 is glycosylated, either with the above described trisaccharide in the 6VW1 system or with the monosaccharide in the 6M0J and 2AJF systems.

In Table S2 is presented the composition of all three simulated systems.

Table S2: The composition of the simulated systems.

	**SARS-CoV-2**				**SARS-CoV**	
	**6VW1**		**6M0J**		**2AJF**	
	# Residues	# Atoms	# Residues	# Atoms	# Residues	# Atoms
ACE2	597	9802	597	9598	597	9673
RBD	194	3070	194	3020	180	2848
H_2O	87382	262146	76599	229797	66380	199140
Na^+		275		241		213
Cl^-		250		218		189
Zn^{2+}		1		1		1
Total		275544		242874		212064

2 Results

2.1 Structural stability of SARS-CoV/-2 in MD simulations

The structural stability of the ACE2 receptor is very similar across the three systems (Fig. S1). Different ACE2 glycosylation states (Table S2) have little influence on the stability of the protein backbone.

One small difference, that is possibly due to the less glycosylated $N90_{\text{ACE2}}$, is visible for the ACE2 residues 70-196 in the 6M0J system. In this region, a long loop followed by the α-helix fluctuates a little more than the rest of the protein. Despite bigger RMSF values, the key binding residues 70-83 still strongly interact with the counterpart RBD.

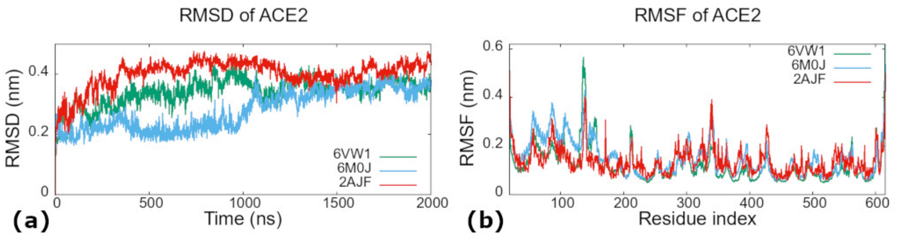

Fig. S1: **Structural stability of the ACE2 receptor in the three simulated systems** throughout 2 μs of MD simulations. (a) The root-mean-square deviation (RMSD) was calculated for the backbone. SARS-CoV-2 systems are represented as green and cyan curves, SARS-CoV is shown in red color. (b) The root-mean-square fluctuations (RMSF) were calculated for C_α atoms.

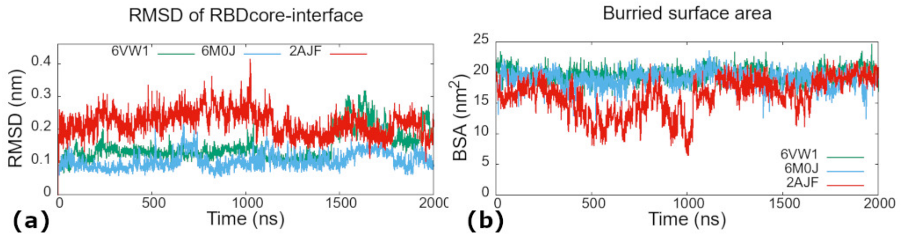

Fig. S2: **ACE2-RBM interface** (a) The root mean square deviation of only the receptor binding motif (RBM; residues 438-508). (b) The size of the RBD-ACE2 interface area as observed during the 2 μs of MD simulation time.

The following graphs clearly show higher stability of the RBD–ACE2 interface in SARS-CoV-2: (i) the RMSD graph for the RBD core residues (N438-P508) directly interacting with the ACE2 (Fig. S2(a)) and (ii) the graph showing the size of the RBD–ACE2 binding surface area (Fig. S2(b)). The average surface area is of 19.6 nm^2 and 19.0 nm^2 for the 6VW1 and 6M0J, respectively, while it is of 16.2 nm^2 for the 2AJF system. Upon visual inspection of the trajectories, to understand why BSA of SARS-CoV decreases between about 400-1000 ns, we saw that during this time a part of SARS-CoV RBD detaches from the ACE2 receptor. The reason why this may be is discussed more in details in the main text.

Table S3: Persistence in time (in %) calculated for Hbonds formed between ACE2 and RBDs of SARS-1 (2AJF) and of SARS-2 (6M0J and 6VW1) during 2 μs of simulation time. The *g_hbond* tool of Gromacs package was used; the distance between Donor and Acceptor atoms were set to less than 3.5 Å and the angle of Hydrogen - Donor - Acceptor was set to less than 30°. ACE2 residues highlighted in red/green/blue background colors belong to the red-K31, green-K353 and blue-M82 binding patterns.

ACE2	SARS-1		SARS-2		
	RESs	2AJF	6VW1	6M0J	RESs
**Q24**	**S461**	-	21	18	**A475**
**Q24**	**N473**	-	18	18	**N487**
**K31**	**N479**	-	21	23	**Q493**
**H34**	**Y440**	12	15	20	**Y453**
**E35**	**N479**	-	64	45	**Q493**
**Y41**	**T486**	7	36	34	**T500**
**Q42**	**Y436**	31	-	-	**Y449**
**Q42**	**Y484**	14	-	-	**Q498**
**Y83**	**N473**	-	86	81	**N487**
**K353**	**G488**	86	74	76	**G502**
**D355**	**T486**	85	56	43	**T500**

2.2 Binding free energy

According to our calculations (Table S4), in all three studied systems, the electrostatic interactions are the driving force for binding and contribute the most to the binding affinity between RBD and ACE2 receptor. The electrostatic, vdW and non-polar contributions are all in favor of SARS-CoV-2 with respect to SARS-CoV.

The MM/GBSA method is known to overestimate the energy values. The electrostatic vacuum energy and the free energy of solvation are very big and nearly cancel each other due to the big differences of the dielectric constants of the vacuum with respect to the one in the solution.

Table S4: The binding free energy $\Delta G_{Binding}$ between ACE2 and RBD of the three systems – 2AJF, 6VW1 and 6M0J – calculated by MM/GBSA method. E_{vdW} and E_{Elec} – the van der Waals and the electrostatic contributions, respectively; G_{polar} – the solvation free energy calculated by Generalized Born; $G_{non-polar}$ – the empirically calculated nonpolar contribution to the solvation free energy. The corresponding standard errors of the mean are noted as well; sd-$\Delta G_{Binding}$ – scaled down-$\Delta G_{Binding}$ using the scale factor of 2.45 suggested for MM/GBSA method [2] (see the main text for details).

Unit: kcal/mol

	E_{vdW}	E_{Elec}	G_{Polar}	$G_{Non-polar}$	$\Delta G_{Binding}$	sd-$\Delta G_{Binding}$
**2AJF**	-66.9 ± 0.3	-604.6 ± 1.3	654.5 ± 1.1	-9.9 ± 0.1	**-26.9 ± 0.3**	**-11.0 ± 0.1**
**6VW1**	-79.8 ± 0.1	-483.1 ± 1.3	543.6 ± 1.2	-11.6 ± 0.0	**-30.8 ± 0.1**	**-12.6 ± 0.1**
**6M0J**	-75.8 ± 0.2	-608.9 ± 1.1	663.1 ± 1.1	-11.5 ± 0.0	**-33.1 ± 0.2**	**-13.5 ± 0.1**

Despite of different drawbacks, the MM/GBSA method is widely used in the scientific community to estimate qualitatively the differences in binding free energies. We have used MM/GBSA results to draw qualitative conclusions on relative binding affinities between SARS-CoV-2 and SARS-CoV viruses, which are in line with other results obtained by different analyses carried out in our study. In addition, they are compatible with the experimental data [3–5].

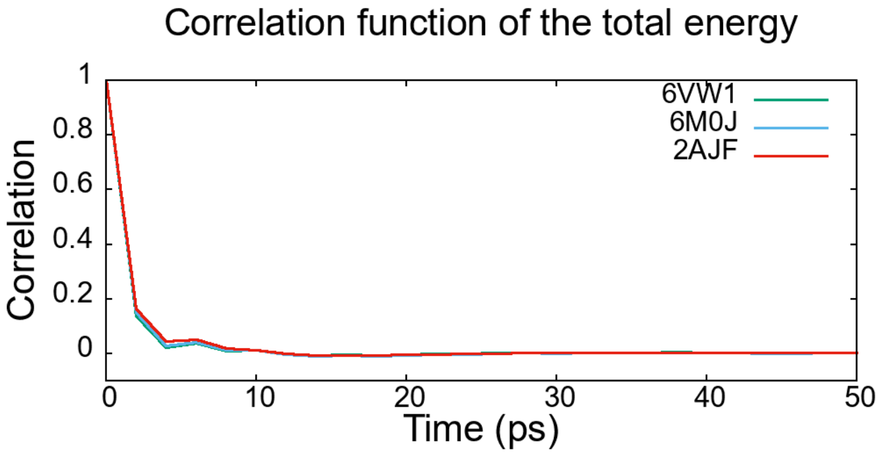

Fig. S3: **The correlation time** for the three studied systems, 2AJF, 6M0J and 6VW1, defined by plotting the auto-correlation function of the total energy from the MD simulation code. We estimate that the correlation time for our systems is about 10 ps, that is much shorter than the time interval of 1 ns that we used to calculate the binding free energies. This result confirms that the binding free energies are non-correlated.

Listing 1: The TCL script finding all pairs of RBD–ACE2 residues that are distant from each other at most 5 Å . It was run in VMD program [6].

```
set fileId [open ''6m0j_ace2.dat" ''w"]

set sel [atomselect top ''not hydrogen and
index 9599 to 12599 and same residue as within 5 of
(index 1 to 9514 and not hydrogen)"]

set n [molinfo top get numframes]
for { set i 0 } { $i < $n } { incr i } {
  $sel frame $i
  $sel update
  set a [$sel get resid]
  set l [llength $a]
  puts $fileId "$i $l $a"
}
close $fileId
################# END #######################
```

Listing 2: The AWK script counting through all MD simulation frames how many times residue A interacts with the residue B and calculating the probability/frequency of the interaction's occurrence. Results obtained by these scripts are shown in Table 1 and Table S3.

```
# How to run:
# sh awk-script2.sh < output-tcl-script1.dat
awk 'BEGIN{ rmax=0 }
{if ($1!="#") {
    for (i=1;i<=$2;i++) {
      r=$(i+2)
      p[r] += 1.0
      if (r>rmax) rmax=r}
    nf++}
}END{
  print ''# residue probability"
  for (r=1;r<=rmax;r++) {
    if (p[r]>0) printf ''%5d %8.3f\n",r,p[r]/nf
}}'
```

References

[1] Nguyen, L.H., Tran, T.T., Truong, L.T.N., Mai, H.H., Nguyen, T.T.: Overcharging of the zinc ion in the structure of the zinc-finger protein is needed for DNA binding stability. Biochemistry **59**(13), 1378–1390 (2020). https://doi.org/10.1021/acs.biochem.9b01055

[2] DasGupta, D., Mandalaparthy, V., Jayaram, B.: A component analysis of the free energies of folding of 35 proteins: A consensus view on the thermodynamics of folding at the molecular level. Journal of Computational Chemistry **38**(32), 2791–2801 (2017)

[3] Walls, A.C., Park, Y.-J., Tortorici, M.A., Wall, A., McGuire, A.T., Veesler, D.: Structure, function, and antigenicity of the sars-cov-2 spike glycoprotein. Cell **181**(2), 281–292 (2020)

[4] Shang, J., Ye, G., Shi, K., Wan, Y., Luo, C., Aihara, H., Geng, Q., Auerbach, A., Li, F.: Structural basis of receptor recognition by sars-cov-2. Nature **581**(7807), 221–224 (2020)

[5] Wrapp, D., Wang, N., Corbett, K.S., Goldsmith, J.A., Hsieh, C.-L., Abiona, O., Graham, B.S., McLellan, J.S.: Cryo-em structure of the 2019-ncov spike in the prefusion conformation. Science **367**(6483), 1260–1263 (2020)

[6] Humphrey, W., Dalke, A., Schulten, K.: Vmd: visual molecular dynamics. Journal of molecular graphics **14**(1), 33–38 (1996)
